# Sport-specific reaction time after dehydration varies between sexes

**DOI:** 10.1186/1550-2783-11-S1-P29

**Published:** 2014-12-01

**Authors:** PH Falcone, C Tai, LR Carson, JM Joy, MM Mosman, JL Straight, SL Oury, C Mendez, NJ Loveridge, JD Griffin, MP Kim, JR Moon

**Affiliations:** 1 Sports Science Institute, MusclePharm, Corp. Denver, Colorado, USA; 2 University of Nebraska, Lincoln, Nebraska, USA; 3 Metropolitan State University of Denver, Denver, Colorado, USA; 4 University of Northern Colorado, Greeley, Colorado, USA; 5 Widener University, Chester, Pennsylvania, USA; 6 United States Sports Academy, Daphne, Alabama, USA

## Background

Dehydration has been shown to decrease sports performance. However, the exact cause of the decreased performance due to dehydration is still unclear. PURPOSE: To compare sport-specific reaction time values between men and women and at different quartiles after a dehydrating protocol to approximately 2% body mass loss.

## Methods

Ten women and eleven men between the ages of eighteen and thirty-five volunteered to participate in the study (27 +/- 4yr, 78.7 +/- 14.8 kg, 174.0 +/- 7.5 cm). Subjects reported to the lab in a fasted and normally hydrated state and completed a two-minute, multi-directional sport-specific reaction time test. Subjects then ran on a treadmill at 80% estimated max HR for 30 minutes, followed by multiple 15 minute sessions in a dry sauna at approximately 150 degrees F. After reaching a 2% (+/- 0.4%) reduction in dry body weight subjects completed the same procedures as pre-dehydration. Reaction times were separated into quartiles (each quartile being a 30-second interval of the two minutes) and averaged to examine the data within each test. Consent to publish the results was obtained from all participants.

## Results

The average total (Q1-Q4) reaction time for men and women combined after dehydration (1375 +/- 210 milliseconds (ms)) was significantly higher than before dehydration (1305 +/- 178 ms; p = 0.0040). The average total reaction time for women after dehydration (1366 +/- 400 ms) was significantly higher than before dehydration (1304 +/- 380 ms; p = 0.0048). However, men did not demonstrate a significant change in reaction time from pre (1305 +/- 300 ms) to post-dehydration (1383 +/- 0.0516 ms; p = 0.066). When quartiles were compared, the average reaction time for women was significantly higher in the third quartile of post-dehydration (1404 +/- 245 ms; p = 0.022) and the fourth quartile of post-dehydration (1412 +/- 263 ms; p = 0.019) than the first quartile of pre-dehydration (1272 +/- 198 ms). Regarding men, the average reaction time was significantly higher in the first quartile of post-dehydration (1427 +/- 220 ms) than the first quartile of pre-dehydration (1285 +/- 149 ms; p = 0.040), the second quartile of pre-dehydration (1285 +/- 189 ms; p = 0.012), the third quartile of pre-dehydration (1338 +/- 200 ms; p = 0.018), and the fourth quartile of pre-dehydration (1312 +/- 236 ms; p = 0.013). Additionally, the average reaction time was significantly higher in the second quartile of post-dehydration (1353 +/- 211 ms) than the first quartile of pre-dehydration (1285 +/- 149 ms; p = 0.046).

## Conclusions

Dehydration appears to affect the sport-specific, total body reaction time performance of athletic men and women differently. Overall, average reaction time was significantly greater after dehydration for the combined group of men and women; however, women’s average reaction time was significantly greater after dehydration, and men’s reaction time trended upward, but did not reach significance. When divided into quartiles, the data suggest that women slowed down (i.e., higher reaction time) within each test and between the pre and posttests, though significance was only observed when comparing the beginning of pre-dehydration and the end of post-dehydration. Regarding quartiles with men, the highest time point was the beginning of the post-dehydration test, and their subsequent reaction times trended lower from quartile to quartile, suggesting improvement. Future studies could include more subjects or a longer test in order to elucidate the discrepancy of these data. Nevertheless, this study suggests that sex differences exist regarding effects of dehydration, such that women’s reaction time performance was significantly affected, whereas men’s performance did not change significantly, due to a possible recovery of performance during the test.

